# Targeting Lymphomas Through MALT1 Inhibition

**DOI:** 10.18632/oncotarget.819

**Published:** 2012-12-31

**Authors:** Lorena Fontan, Ari Melnick

**Affiliations:** Department of Medicine, Weill Cornell Medical College, New York, NY; Department of Medicine, Weill Cornell Medical College, New York, NY

Diffuse large B-cell lymphoma (DLBCL), the most common form of non-Hodgkin Lymphoma, comprises a heterogeneous group of diseases that can be classified into at least three different entities with distinct gene expression signatures. Among these, the activated B cell-like (ABC)-DLBCLs are the most resistant to current standard immune-chemotherapy regimens [1]. Development of novel targeted therapies with activity in this DLBCL subtype is required to improve clinical outcome for these patients.

**Figure 1:**
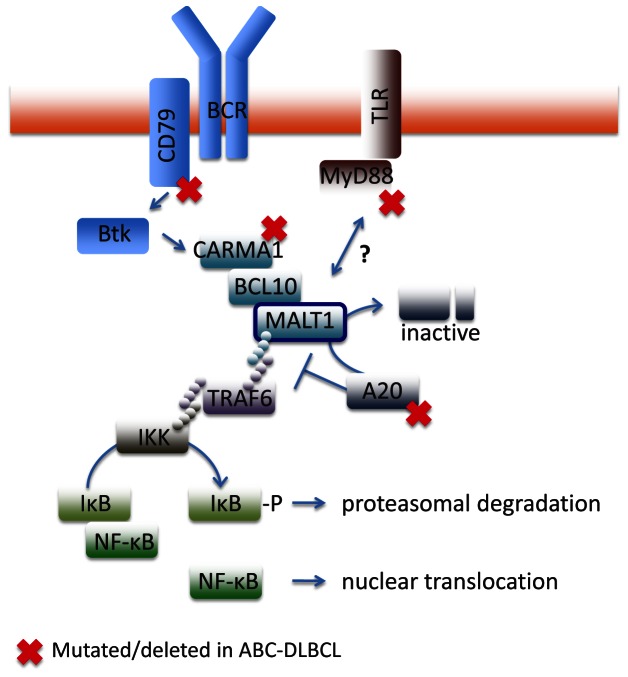
Somatic mutations leading to ABC-DLBCL Chronic BCR or TLR signaling dependent on activating mutations of CD79A/B, CARMA1 or MYD88 or on A20 inactivation through mutation or deletion trigger NF-κB activation and ABC-DLBCL survival and proliferation. MALT1 is a mediator of NF-κB activation and indirectly sustains this activation by inhibiting the NF-κB signaling attenuation mechanisms, A20 and others. Targeting of MALT1 protease activity impairs lymphoma survival by inhibition of NF-κB signaling, leading to growth inhibition and apoptosis in xenograft models of ABC-DLBCL.

Efforts to identify recurrent somatic mutations in ABC-DLBCLs have revealed common biological themes despite their considerable genetic heterogeneity. Most notably frequent activating somatic mutation in B-cell receptor (BCR) and Toll like receptor (TLR) signaling pathways, leading to constitutive NF-κB activity [2]. Along these lines the MALT1 paracaspase has emerged as a key signaling mediator and therapeutic target in ABC-DLBCLs due to its role in transducing signals from several receptors implicated in lymphomagenesis [3]. To carry out its functions MALT1 assembles into a complex with CARMA1 and BCL10 (CBM complex), which facilitates TRAF6 induced activation of IKK and p65/c-Rel signaling (Figure 1) and triggers MALT1 protease activity. MALT1 is distinct from other caspases; it cleaves after Arginine residues and targets different substrates. Substrates of MALT1 include the ubiquitin editase A20/TNFAIP3, the deubiquitinase CYLD, the MALT1 binding protein BCL10 and the NF-κB subunit RelB [3]. Cleavage of these proteins enables NF-κB activation ultimately promoting cell survival and proliferation [3]. Likely MALT1 may cleave additional proteins yet to be discovered, with potential implications in various pathways and biological processes.

The *MALT1* gene itself is affected by translocations in certain mucosa associated lymphoid tissue lymphomas that may either lead to its deregulated expression or to gain of function activities [3]. More specifically, API2-MALT1 fusion results in a gain of function cleavage of the kinase NIK by constitutively activated MALT1, leading to aberrant induction of the non-canonical NF-κB pathway [3].

Its location downstream of multiple lymphomagenic pathways makes MALT1 an excellent therapeutic target. Along these lines, a peptide that can bind and inhibit the MALT1 catalytic pocket (Z-VRPR-FMK) was shown to downregulate NF-κB activity, suppress proliferation and induce apoptosis in ABC-DLBCL cell lines known to contain mutations in TLR and BCR signaling components [4, 5]. These data revealed that MALT1 is a bona fide therapeutic target, inhibition of which may disrupt oncogenic signaling induced by somatic mutations in ABC-DLBCLs. However Z-VRPR-FMK is not suitable as a therapeutic agent due its unfavorable pharmacological properties.

Fortunately, two recent studies identified small molecule inhibitors of the MALT1 protease activity with potent activity against ABC-DLBCL cells both *in vitro* and *in vivo* [6, 7]. One of the studies identified phenothiazine compounds as mediating reversible MALT1 cleavage inhibition [7]; whereas the other identified a novel compound termed “MALT1 inhibitor-2” (MI-2), that irreversibly binds the active site of MALT1 and potently suppresses its enzymatic activity at nanomolar concentrations [6]. Irreversible inhibition of MALT1 by MI-2 is quite appealing since this enables its effect to be prolonged over time allowing sustained inhibition of its downstream pathways. It is notable ABC-DLBCL cell lines with mutations in the BCR pathway upstream of MALT1 (CD79A/B, CARMA1) or the TLR pathway (MyD88) were sensitive to MALT1 cleavage inhibition [6]. In contrast cell lines with mutations downstream of MALT1 exhibited impaired response to MALT1 cleavage inhibition [6] suggesting that these should be considered possible resistance mechanisms. Along these lines Nagel et al. demonstrated that MALT1 cleavage inhibition resistance can be induced in cell lines by overexpression of a constitutively active form of IKKβ that activates NF-κB downstream of MALT1 [7]. Hence, the rational translation of MALT1 inhibitors to the clinic will require the identification of the mutations stimulating aberrant NF-κB signaling so that the proper therapeutic agents can be selected to enable maximal efficacy and minimize tumor resistance.

Inhibition of the BCR signaling mediator BTK (Bruton Tyrosine Kinase) has emerged as another promising therapeutic approach for ABC-DLBCLs. The irreversible BTK inhibitor Ibrutinib has potent activity in pre-clinical models and promising activity in early phase clinical trials [8]. However not all ABC-DLBCLs will benefit from BTK inhibition. For example CARMA1 mutations activate the CBM complex downstream of BTK and hence render lymphomas unresponsive to BTK inhibitors, whereas MALT1 inhibitors are still effective in the presence of CARMA1 mutations. Because recent data indicate that other B-cell lymphomas are also highly addicted to BCR signaling and may be sensitive to inhibitors like Ibrutinib (i.e. such as chronic lymphocytic leukemia), it is likely that MALT1 inhibitors will be useful beyond ABC-DLBCLs and MALT lymphomas. Finally, combination of MALT1 and BTK inhibitors or other targeted anti-lymphoma therapies or chemotherapy needs to be evaluated to provide maximal suppression of oncogenic BCR signaling, as well as to suppress complementary pathways that might enable lymphoma cell survival (i.e. such as BCL6 and BCL2) and potentially foster the emergence of resistant clones of lymphoma cells. .
